# The MacGyver effect: alive and well in health services research?

**DOI:** 10.1186/1472-6963-11-226

**Published:** 2011-09-20

**Authors:** Roshan Perera, Helen J Moriarty

**Affiliations:** 1 Medical Education Unit, Department of the Dean, School of Medicine and Health Sciences, University of Otago, Wellington, P.O. Box 7343, Wellington South 6242, New Zealand; 2 Department of Primary Health Care and General Practice, School of Medicine and Health Sciences, University of Otago, Wellington, New Zealand

## Abstract

**Background:**

In a manner similar to the television action hero MacGyver, health services researchers need to respond to the pressure of unpredictable demands and constrained time frames. The results are often both innovative and functional, with the creation of outputs that could not have been anticipated in the initial planning and design of the research.

**Discussion:**

In the conduct of health services research many challenges to robust research processes are generated as a result of the interface between academic research, health policy and implementation agendas. Within a complex and rapidly evolving environment the task of the health services researcher is, therefore, to juggle sometimes contradictory pressures to produce valid results.

**Summary:**

This paper identifies the MacGyver-type dilemmas which arise in health services research, wherein innovation may be called for, to maintain the intended scientific method and rigour. These 'MacGyver drivers' are framed as opposing issues from the perspective of both academic and public policy communities. The ideas expressed in this paper are illustrated by four examples from research projects positioned at the interface between public policy strategy and academia.

## Background

Angus 'Mac' MacGyver was a secret agent, hero of an ABC action-adventure television series that ran from 1985-1992. MacGyver was a troubleshooter who used his scientific training and existing resources in a creative way, to create simple albeit ingenious solutions to overcome unexpected problems or to resolve difficult situations. His trademark was to be resourceful and innovative. In one episode the hero described his drive for resourcefulness in the following quote "...the tighter your plan the more likely you are to run into something... unpredictable"[1]. The eponymous 'MacGyver Effect' has come into common parlance since the 1990's as a result of the popular television series. The MacGyver Effect is the ability to apply scientific principles and use everyday things in an innovative way to create what is needed to overcome obstacles thrown into the path by another party. This paper sets out to show how the MacGyver Effect operates within health services research.

Health services research is a "multidisciplinary field of scientific investigation that studies how social factors, financing systems, organizational structures and processes, health technologies, and personal behaviours affect access to health care, the quality and cost of health care, and quantity and quality of life"[2]. Health services research programmes are often triggered by strategic service or public policy shifts and are therefore undertaken concurrently with actual or impending changes to the health services being researched. Two important features distinguish health services research: the need to ensure that research outputs are relevant (at both policy and practice levels) and expediency [3].

Academic research processes inevitably converge with health service or health policy work streams during the conduct of health services research and this generates complicating imperatives. Emerging issues call for urgent responses and under these circumstances the merger of academic research and health sector initiatives or new policy work streams may be both reasonable and prudent. Indeed the research itself may be funded under a policy imperative, and the research process may then assume immediate significance in a 'real world' context.

However, blending research and public policy is not always a smooth process. Planned research methods may be subsumed by, curtailed for, or adapted to health service implementation requirements. Resourcefulness and innovative approaches are often required, in order to deal with the unpredictable arising from seemingly well circumscribed research plans, or to find pragmatic solutions to complex theoretical dilemmas while also ensuring the rigour of the research undertaken.

This paper identifies the challenges to robust research processes which can appear, seemingly unpredictably, as a result of the interface between academic research, health policy, and service and consumer agendas. In particular, the paper focuses on three main drivers that these authors deem responsible for MacGyver type dilemmas in health services research. The challenges generated by each 'MacGyver driver' are discussed to outline and make explicit the underlying assumptions and approaches to manage differences in viewpoint between academic and public policy communities, and to stimulate debate on these issues. The authors postulate that these drivers are likely to be common to many other research projects situated in a key health service research setting, which may be politically sensitive, or otherwise controversial.

It would be true to state that researchers don't always produce quality research in the absence of external constraints. However, the impact of external constraints on well constructed and scientifically sound methodology and ethically approved research processes can undermine one or more of those attributes. Pressure to curtail parts of agreed research methods, adjust the focus of analysis, or alter the nature of the reporting to meet a new agenda of the funder or other external party, may carry the potential to seriously jeopardise the validity of the research. These arguments may, however, have a very small impact on funders who have different goals or wish to demonstrate some action within an area of contention.

Reflection on the issues which arose during the separate research experiences of each of the authors has facilitated the identification of the MacGyver drivers. In this paper, four health services research projects in which the authors participated are thus used to illustrate the MacGyver drivers and resulting challenges.

In each of the given research examples there has been a need to argue for maintaining the agreed research methodology and/or analysis and reporting of results.

Each of these example projects was highly controversial at the time, and was positioned at the interface between public policy strategy and academia. Each provided the opportunity for a MacGyver-style approach to deal with specific circumstances and difficulties. In some examples this opportunity was realised through pragmatic compromise. In others, researchers used innovative thinking to create new tools and methodologies; or forged on with original research plans in the face of adversity whilst anticipating and preparing for resultant consequences.

## Discussion

### The challenges of health services research

MacGyver-type dilemmas arise in health services research due to three main drivers:

1) Differences in organisational 'culture' such that the research institution and the health service provider or funder do not necessarily respect each other's requirements; 

2) Time and timeliness constraints imposed on the research by public policy implementation demands; and

3) Intellectual property and ownership limitations.

Innovative thinking may be called for to ensure that these drivers do not derail the research programme, or indeed threaten the underlying scientific method and rigour.

Each of these 'MacGyver drivers' is discussed in more detail below, framed as opposing issues from a health services researcher and a sector/policy perspective.

#### 1) Culture Clash

A lack of accord between research and public policy perspectives can occur during health services research projects, largely due to differences between policy and research 'cultures'. The importance of organisational culture, and existence of differences in the world views of academics, funders and policy makers has been previously described [4-6]. The practical impact of these differences in the execution of health services research processes and outputs is described below.

##### a) Implicit v explicit agendas

**Figure 1:**
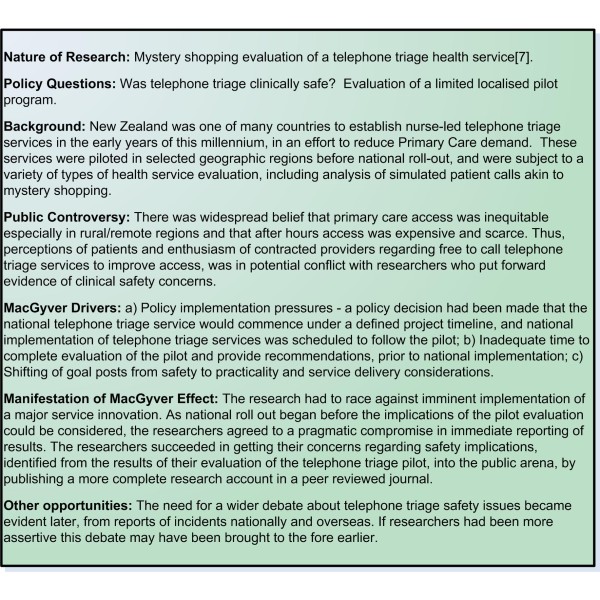
**Example 1 Telephone triage evaluation**.

The role of research is to maintain explicit, transparent and replicable processes, while often policy and service agendas are implicit rather than overt, or are driven by political expediency and goal setting. Implicit or hidden policy or service delivery agendas may result in only partial communication of desired objectives, and impact on clarity of messages relayed to researchers with respect to the research outputs required. Additionally, unexplained shifting of 'goal posts' partway through the research processes, may result in stakeholder dissatisfaction with final outputs, as well as potentially creating confusion, and further compressing already tight time frames. The 'goal posts' for telephone triage evaluation [7] for example, became one of service delivery rather than clinical safety, and the researchers felt that their safety concerns were unheeded (Figure 1).

Other manifestations of hidden policy or service agendas include the initiation of alternate but concurrent work streams as a risk management tactic. Such tactics may be driven by impatience with the research timeline or scientific process, misunderstanding of innovation, and intolerance of the need to understand underpinning theory and interpret empirical results. This may result in parts of the planned programme of research being handed over to others, possibly even non-researchers, to complete. There may sometimes be little communication with researchers about the introduction, by the funding body, of such additional siloed work streams, particularly with regard to explanations about what is to be done separately, who is involved or why.

##### b) Selective reporting and implementation

Research ethics promote a comprehensive approach to conveying results in contrast to policy and implementation imperatives which may encourage selective reporting of research outputs, or an 'appreciative' explanation of otherwise unacceptable findings.

In certain situations where the results of the health services research may not be palatable for the funding body, it is possible that only some of the recommendations in reports submitted may be accepted in the implementation process. Despite professed impartiality, the realisation that the research record will become a public record often entails a degree of nervousness on the part of policy implementation partners. This may particularly relate to information that would be included in research reports. Mixed messages are therefore sometimes apparent from policy implementation partners during the preparation of research outputs, with professions of laudable albeit sometime elusive impartiality, suggestions bordering on instructions, and a sense of seeking to reject advice before it has been fully delivered.

In two examples, the extent of disclosure of results needed to be addressed in consultation between the research team and the funding body, to ensure that the research results were presented in a form that would satisfy both the academic and the service perspectives.

**Figure 2:**
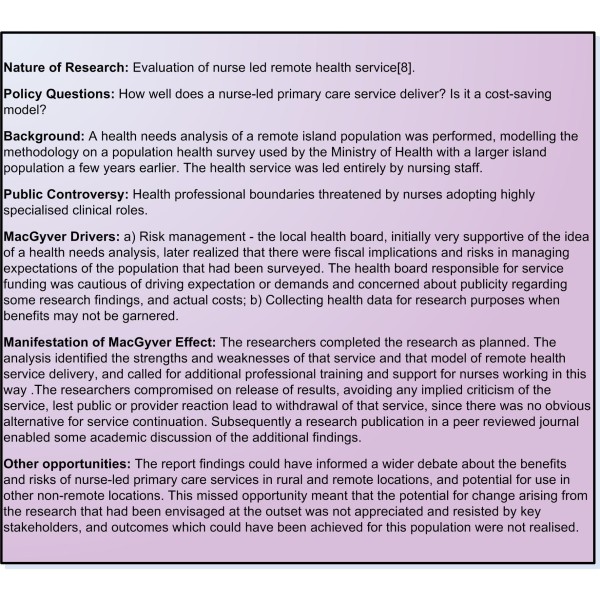
**Example 2 Nurse led remote health service evaluation**.

In reporting on an evaluation of a remote community-based health service (Figure 2) care had to be taken to emphasise the positive and explain away the weaker aspects of service elements that had been identified, but were not readily amenable to change. Publicity about health service weaknesses could create anxiety amongst the population and possibly drive expectations and demands that could not be met [8].

**Figure 3:**
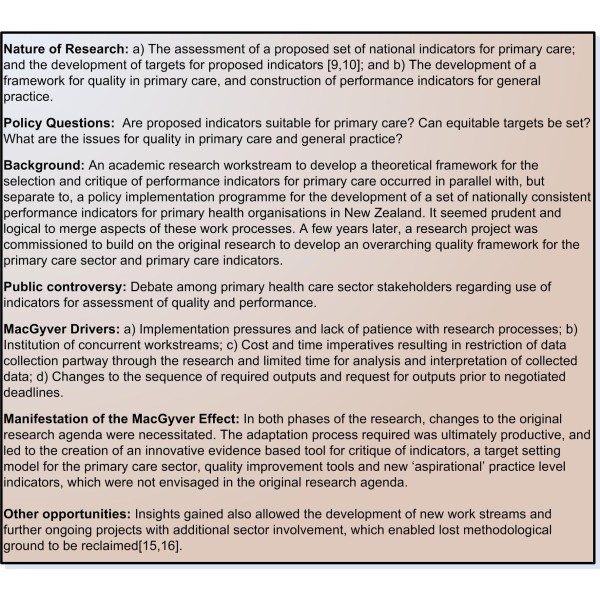
**Example 3 Assessment of indicators and quality in primary health care**.

During commissioned research for the assessment of suitability of a set of national indicators for primary care (Figure 3) there was significant initial concern from policy implementers with regard to inclusion in the final report, of representative quotes from qualitative interview data about potential indicators, and interpretation of what constituted a 'fail' for assessed indicators with only some recommendations being fully accepted [9,10].

It could be argued that selective implementation by funders could not be called a lapse in impartiality given that academic researchers are advisors to, not directors of, policy. Nevertheless from the academic perspective, it highlights the necessity of learning how to deal with the occasions when the principles behind the selective implementation decisions of policy or service partners remain unclear.

##### c) Risk management by policy or service funders

The MacGyver driver of 'risk management' is most often pervasive during commissioned health services research projects, even when the nature of the risk, the direction it is coming from and the consequences of not managing that risk are unclear. This may result in restriction of access for health services researchers, to required but siloed information, causing frustration on the part of the researchers, contrasted with defensiveness and fear on the part of the public policy/health service provider community of possible repercussions from undue publicity.

From the public policy or provider perspective risk management may occur in order to protect certain provider or funder sectors from implicit or perceived criticism, to explain time and fiscal resource constraints, and 'sell' the benefits of change or innovation. In contrast, research institutions may worry about misrepresentation, and prefer to take a 'warts and all' transparency approach, wishing to present the research complexity and uncertainty in all its glory.

**Figure 4:**
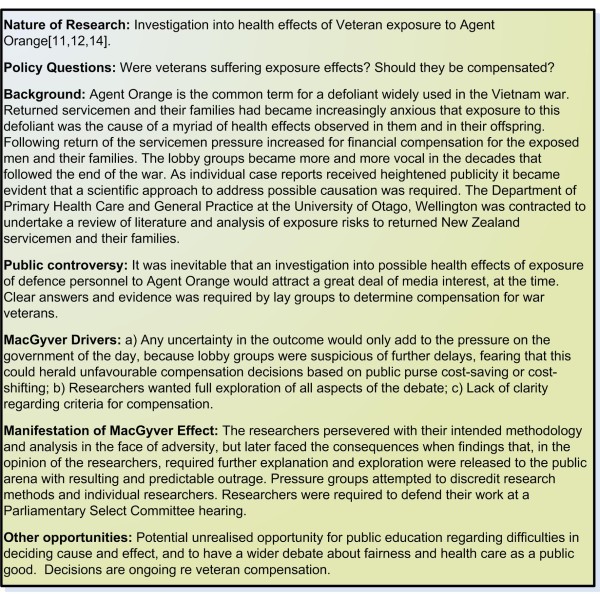
**Example 4 Health effects of exposure to Agent Orange**.

Risk management considerations resulted, in the research team needing to defend their findings in response to pressure groups with regard to the research on Agent Orange (Figure 4) [11], and a requirement for that team to make a subsequent appearance at a Parliamentary Select Committee [12].

So what would MacGyver do? Options for managing these dilemmas might include emphasising the difference in culture, wining public opinion over to his side or simply waiting for a change of circumstances. The tactics of engaging public opinion are generally avoided by researchers, who regard this as unethical behavior, but harvesting public opinion is core business for politicians and that particular culture clash can provide an uneven playing field, where the research team is disadvantaged.

#### 2) Time and timeliness

Time is often the biggest obstacle confronting the health services researcher. Collecting rigorous data sets may be time consuming, and in addition, time-lines often slip for reasons beyond researcher control. Delays may also occur as a result of the need to establish research partners, memoranda of understanding, and negotiation of contracts.

Political pressure for expediency and other expediency drivers (such as funder, provider or service client demands) can appear to reflect lack of respect for the patience needed to conduct research processes. There may be low tolerance from the implementers for delays, lack of regard to researcher claims of complexity, and researchers may face both reasonable and unreasonable external demands to curtail parts of the research agenda. During the second phase of research in example 3 (Figure 3), cost and time imperatives imposed unexpectedly by the funder partway through the research process, required restricting both the extent of data collection and analysis and interpretation of collected data.

Inevitably, time imperatives and real world needs require sector/policy management to provide a simple solution to a complex problem. This, however, leaves the theory underpinning the research in limbo, and unresolved theoretical dilemmas may either be wrongly simplified, or remain hidden each with potentially grave implications for implementation.

Policy implementation imperatives can also prompt demands for early cessation or implementation of outputs before results are obtained or verified. This may lead to premature release of incomplete research findings, or delayed or selective release.

From an academic perspective, omission of research safety checks along the way in the interests of time (for example, not achieving theme saturation, omitting stakeholder feedback loops, not seeking input from peer review or sector commentators) creates the danger of substituting short term gains for longer term risk. Also, ironically, rushed implementation due to impatience with orderly preparation of empirical or theoretical underpinning often risks 'throwing the baby out with the bathwater'. Potential useful innovation may be discarded as irrelevant, or labeled unusable due to inadequate preparation of users or implementation in a piecemeal fashion, as in example 3 (Figure 3), thus wasting both potentially valuable outputs and the time and money spent on undertaking the work. Innovative thinking may therefore be called for to achieve a compromise to maintain academic discipline and research rigour while satisfying implementation imperatives.

The business and management literature notes that the influence of time pressures on creativity is generally negative [13]. However, in health services research, the need for timely resolution of health sector issues serves as a means of facilitating the MacGyver Effect, stimulating the development of pragmatic and innovative solutions.

So what would MacGyver do? Options include early negotiation of the degree to which the academic and research ideal can be retained, premature cessation of the research programme, agreement to intermediate targets, integration of action research methodologies, collaboration with any alternate work streams set up alongside the research process, and decision on allowing a partially evaluated piece of work through while still working towards clarifying the complete picture.

#### 3) Independence vs. intellectual ownership

Preservation of academic autonomy, safeguarding of intellectual property rights and retention of the right to publish any and all results obtained are necessary to maintain academic credibility and the goodwill and confidence of the health care sector. On the Agent Orange saga, Dr Deborah McLeod commented that "Intellectual freedom is one of the cornerstones of academia and, as such, the academic viewpoint is seen to provide good quality and unbiased information"[14].

Nevertheless, when undertaking commissioned research there is often an intrinsic tension with respect to the extent to which the research can be seen to be independent of the funding body. There may also be an obligation to protect data deemed sensitive or potentially damaging unless released with heavy caveats, or even embargoed. Additionally, limitations imposed on the methodology due to the pragmatic considerations mentioned earlier often mean that while the research results obtained are not wrong, they may not be proven to an academic standard, constraining the ability to publish.

The solution for researchers is often to channel garnered knowledge and tools into further research projects to revisit concepts and enable rigour to be added to the original message [15,16]. The addition of extra work streams and personnel however, does also have repercussions on publication and authorship of original work, and ongoing use of unpublished or partially published work, to the detriment of its original creators.

Identification of additional questions may also result in the initiation, by sector partners, of concurrent but separate work streams directed at the same public sector initiative. The major risk in this instance is that use of data sets or tools developed for one purpose may be redirected or utilised by the funder for an allied but different purpose.

Commissioned research outputs, even if incomplete, may also be used by funders for purposes other than that originally intended, and possibly even without acknowledgement of the provenance of the original work. This raises additional tensions and questions especially if the original research activity is brought to a close prematurely.

Our hero MacGyver would inevitably win the day despite unpredictability. Researchers, however, may not always be on a winning streak. It would not make for riveting viewing if MacGyver agreed to an embargo instead of finding a creative alternative solution. Options for MacGyver may, therefore, include inventing new channels into which the knowledge and tools can be used to add rigour; insisting on acknowledgement of provenance; or safeguarding original data for future publication or later use as intended.

### Innovation to maintain relevance

The nature of health services research means that alignment of work processes between academia and public sector/policy is frequently necessary. Health services researchers must interface with both the changing health services and with the agents of change to do the research and ensure relevance of research outputs. Research work programmes can thus become subject to, or be driven by, implementation imperatives. The health services research arena can then become crowded with key players and conflicting agendas, as well as changeable conditions and timeframes. Within this complex and rapidly evolving environment, the task of the health services researcher is to juggle sometimes contradictory pressures to persevere with the scientific method to produce valid results.

In a rapidly changing health care environment maintaining the relevance of the research or 'catching the boat' is vital from both the perspectives of academic research teams and the health or policy sector. Researchers carrying out a rigorous methodology carry a risk of being too slow for policy needs and may be left behind by the turn of events: still doing research into something of theoretical value, but knowing that this may no longer be implementable, and only of interest in an academic sense. Researchers left behind by policy imperatives may be accused of living in an ivory tower, or worse still of having 'missed the bus'. Further, there may be some inappropriateness in carrying out health services research that is not synchronized to real world timeframes.

In true MacGyver form, policy implementation requirements force health services researchers to adapt to circumstances. Thus despite difficulties encountered and changes to the planned research agenda, the resultant dynamic tensions and the adaptation process required can be ultimately productive. Improvised, positive and successful outcomes may result from health services research in these situations. The pressure of unpredictable demands and events and constrained time frames, requires researchers to consider novel alternatives and create tools that are an important innovation and research output in their own right. The results are often both innovative and functional, with the creation of outputs that could not have been anticipated in the planning and design of steady research processes. As a result of the twin demands of policy implementation pressures and the drive to produce results for funders in a timely fashion an innovative tool for indicator critique and an equitable target setting model for primary care were created, which was not envisaged in the original research agenda.(Figure 3)[9,10].

However, in contrast to MacGyver, taking the decision to go for policy or sector 'glory' sometimes means researchers risk potential academic ignominy. The challenge of collaborative contract policy and/or health sector research uses the MacGyver Effect to its full potential: creating rewarding and useful solutions with material to hand. Such solutions are frequently robust, but do not have the backing of the proven rigour of a standard academic approach, as a result of time and other constraints.

Consequently, at many points during projects academic researchers grapple with two unenviable alternatives: the risk of being irrelevant in the short term (because of the wish to work carefully); or of being irrelevant in the long term (because of not being able to work as carefully as necessary to maintain academic rigour).

Collaboration may, therefore, often seem to be a poor substitute for maintenance of academic discipline. The mild mannered and principled innovator that is the MacGyver persona may be a fitting description of health services researchers. Yet, while productive, the MacGyver Effect sits somewhat uncomfortably on academic researchers trained in methodological process.

From an academic perspective it can seem difficult to justify the institution of practice before theory is properly developed. There are longer term risks to validity of the research, and potential consequences of the limitations imposed by taking action based on hurried health services research or incomplete analysis of results obtained. Nevertheless, in an environment where expediency is paramount, the MacGyver Effect may ultimately be the solution to the challenge of health services research.

## Conclusion

This paper has characterized some of the challenges inherent in conducting health services research as a result of opposing drivers between academic and public policy/health sector communities - the focus for academics on process, and sector/policy on outputs. We freely acknowledge that the complexity and conflict that this engenders is not readily amenable to change. However, each player needs to determine the extent to which he or she can live with the results. Researchers should be alert to the potential for MacGyver Drivers in their own research and question if the MacGyver Effect may have exerted an influence on health services research now completed and published.

MacGyver would seem to have been in his element if he had made his career in health services research, rather than as a secret agent.

## Summary

Academic research processes inevitably converge with health service or health policy work streams during the conduct of health services research.

The blending of research and public policy does not always work smoothly.

MacGyver-type dilemmas arise in health services research due to three main drivers. Innovation may be called for to ensure that these drivers do not derail the research programme, or indeed threaten the underlying scientific method and rigour.

These 'MacGyver drivers' are:

1) Differences in organisational 'culture' such that the research institution and the health service provider or funder do not respect each other's requirements;

2) Time and timeliness constraints imposed by public policy implementation demands; and

3) Constraints of intellectual property and ownership limitations.

In true MacGyver form, policy imperatives may also be a positive influence in research. Policy implementation imperatives may force researchers to consider innovative alternatives and create tools that are an important innovation and research output in their own right. Thus, despite difficulties encountered and changes to the planned research agenda, the resultant dynamic tensions and the adaptation process required can be ultimately productive.

The challenges inherent in conducting health services research are a result of opposing drivers between academic and public policy/health sector communities - the focus for academics on process, and sector/policy on outputs. We freely acknowledge that the complexity and conflict that this engenders is not readily amenable to change.

The MacGyver Effect sits somewhat uncomfortably on academic researchers trained in methodological process. But in an environment where expediency is paramount, the MacGyver Effect may ultimately be the solution to the challenge of health services research. Each player needs to determine the extent to which he or she can live with the results.

## List of Abbreviations used

ABC: American Broadcasting Company.

## Competing interests

The authors declare that they have no competing interests.

## Authors' contributions

This paper was co-written by both authors. Both authors were involved in all stages of the paper from drafting to completion, and the paper draws on the separate research experiences of each author. Both authors have read and approved the final manuscript.

## Pre-publication history

The pre-publication history for this paper can be accessed here:

http://www.biomedcentral.com/1472-6963/11/226/prepub
